# Discharge or admit? Emergency department management of incidental pulmonary embolism in patients with cancer: a retrospective study

**DOI:** 10.1186/s12245-017-0144-9

**Published:** 2017-06-06

**Authors:** Srinivas R. Banala, Sai-Ching Jim Yeung, Terry W. Rice, Cielito C. Reyes-Gibby, Carol C. Wu, Knox H. Todd, W. Frank Peacock, Kumar Alagappan

**Affiliations:** 1 Department of Emergency Medicine, The University of Texas MD Anderson Cancer Center, 1515 Holcombe Boulevard, Unit 1468, Houston, TX 77030 USA; 2 Present address: Emergency Department, Caboolture Hospital, McKean Street, Caboolture, Queensland 4510 Australia; 3 Department of Diagnostic Radiology – Thoracic Imaging, The University of Texas MD Anderson Cancer Center, 1515 Holcombe Boulevard, Unit 1478, Houston, TX 77030 USA; 4 Present address: EMLine.org, Mendoza, Argentina; 5 Department of Emergency Medicine, Baylor College of Medicine, Houston, TX 77030 USA

**Keywords:** Incidental pulmonary embolism, Cancer, Emergency, Outpatient

## Abstract

**Background:**

Hospitalization and early anticoagulation therapy remain standard care for patients who present to the emergency department (ED) with pulmonary embolism (PE). For PEs discovered incidentally, however, optimal therapeutic strategies are less clear—and all the more so when the patient has cancer, which is associated with a hypercoagulable state that exacerbates the threat of PE.

**Methods:**

We conducted a retrospective review of a historical cohort of patients with cancer and incidental PE who were referred for assessment to the ED in an institution whose standard of care is outpatient treatment of selected patients and use of low-molecular-weight heparin for anticoagulation. Eligible patients had received a diagnosis of incidental PE upon routine contrast enhanced chest CT for cancer staging. Survival data was collected at 30 days and 90 days from the date of ED presentation and at the end of the study.

**Results:**

We identified 193 patients, 135 (70%) of whom were discharged and 58 (30%) of whom were admitted to the hospital. The 30-day survival rate was 92% overall, 99% for the discharged patients and 76% for admitted patients. Almost all (189 patients, 98%) commenced anticoagulation therapy in the ED; 170 (90%) of these received low-molecular-weight heparin. Patients with saddle pulmonary artery incidental PEs were more likely to die within 30 days (43%) than were those with main or lobar (11%), segmental (6%), or subsegmental (5%) incidental PEs. In multivariate analysis, Charlson comorbidity index (age unadjusted), hypoxemia, and incidental PE location (*P* = 0.004, relative risk 33.5 (95% CI 3.1–357.4, comparing saddle versus subsegmental PE) were significantly associated with 30-day survival. Age, comorbidity, race, cancer stage, tachycardia, hypoxemia, and incidental PE location were significantly associated with hospital admission.

**Conclusions:**

Selected cancer patients presenting to the ED with incidental PE can be treated with low-molecular-weight heparin anticoagulation and safely discharged. Avoidance of unnecessary hospitalization may decrease in-hospital infections and death, reduce healthcare costs, and improve patient quality of life. Because the natural history and optimal management of this condition is not well described, information supporting the creation of straightforward evidence-based practice guidelines for ED teams treating this specialized patient population is needed.

## Background

Venous thromboembolism is a condition that includes both deep vein thrombosis and pulmonary embolism (PE). Acute PE is a potentially life-threatening medical emergency that demands urgent intervention [1–3]. Although the signs and symptoms that typically herald PE can range from subtle to severe [4, 5], it is possible that a PE will produce no signs or symptoms at all. An increasing number of PEs are being detected incidentally in otherwise asymptomatic patients, such as those undergoing chest computed tomography (CT) for an unrelated purpose. With the advent of multi-row detector CT scanners, even small emboli in subsegmental arteries can now be detected [6].

Immediate anticoagulation therapy remains the standard of care for PE, as any delay may increase the risks associated with this life-threatening condition [7, 8]. Although patients with symptomatic PE have traditionally been admitted to hospital to initiate anticoagulation and to avoid complications secondary to PE [1], recent studies suggest that selected patients could be treated successfully on an outpatient basis [9, 10]. For a PE that is discovered incidentally, however, the optimal therapeutic strategies are less clear [11]; the current recommendation is that incidental/asymptomatic PE should be treated with anticoagulation therapy, just as for symptomatic PE [12, 13].

Decisions about optimal management of an incidental PE become more complicated when the patient also has cancer [14]. The hypercoagulable state associated with malignancy makes venous thromboembolism particularly prevalent in these patients [6], even when the cancer is newly diagnosed [4, 15]. Treatment of cancer patients is often complicated by confounding comorbidities, such as thrombocytopenia, intracerebral metastases, and friable or bleeding tumors. Mortality from an acute thrombotic event is four to eight times greater in patients with cancer than in those without cancer [16–18], and evidence of venous thromboembolism has been reported in as many as half of patients with cancer at the time of postmortem examination [13].

Emergency providers have noted that incidental PE is a frequent reason for presentation by patients with cancer [19], who increasingly are utilizing emergency departments (EDs), including general EDs, for acute care [20]. Current guidelines [12] consider patients with cancer and PE as too high-risk for safe discharge from the ED. Thus, especially in general EDs, cancer patients with PE are usually admitted to the hospital, even though several professional societies [2, 12, 21, 22] recommend outpatient management for selected patients [23]. Given that there were more than 14 million cancer survivors in the USA in 2015 [24]—and an expected 18 million by 2022 [25]—and that the reported proportion of cancer patients incidentally diagnosed with PE during CT is not negligible, ranging from approximately 1 to 4% [11, 12, 26, 27], ED treatment teams are increasingly in need of straightforward, evidence-based practice guidelines for this specialized patient population.

Because the perceived high risk associated with cancer and coincident PE makes the performance of a prospective study challenging, we designed a retrospective study to investigate whether there is a subset of cancer patients for whom discharge might be safe and thus the consideration of a prospective study be warranted. If such a cohort could be identified, then future study in an environment more generalizable to a routine ED population could be pursued. The purpose of our study was to describe outcomes in cancer patients with a diagnosis of incidental PE who were referred to the ED in a comprehensive cancer center. We compared outcomes by PE location, Charlson comorbidity age-unadjusted index, cancer type and stage, functional status, and disposition (discharged home with anticoagulant therapy versus admitted to the hospital). We expected that outpatient treatment of suitable patients would not be associated with poorer survival outcomes.

## Methods

### Study design

This study is a historical cohort design with data collected via chart review of outpatients who were seen in the The University of Texas MD Anderson Cancer Center ED between January 1, 2013 and December 31, 2014 pursuant to a diagnosis of incidental PE. Demographic, clinical, and survival data were abstracted retrospectively from the electronic medical record.

### Study setting and population

#### Setting

MD Anderson is a comprehensive cancer center in Houston, TX. Its unique assets, including a high volume of cancer patients and an ED that serves them almost exclusively, provide an excellent environment in which to study this group of patients. The MD Anderson ED has 43 beds and is staffed by physicians from the institution’s Department of Emergency Medicine, the first academic department of emergency medicine to be founded in a comprehensive cancer center. The ED provides care to patients with acute needs who are either too sick to be safely or comfortably treated in clinic or who need after-hour care, more than 95% of whom are currently receiving cancer treatment or are survivors. In 2015, the ED handled more than 28,100 patient visits from 14,800 unique patients, an average of more than 75 patients per day. Services include care for treatment-related side effects, disease progression, and comorbidities.

At MD Anderson, patients whose staging CT identifies an incidental PE are frequently referred to the ED for evaluation, either due to the timing of the diagnosis or to obtain an evaluation that is more thorough or time consuming than can be provided in the outpatient clinic. The frequency of this occurrence is so significant that patients have been designated in the electronic medical record as having “incidental PE on CT” as their chief complaint. Emergency physicians play a central role in choosing the initial management strategy for these patients. In most cases, ED physicians function independently of oncologists regarding initial management, treatment, and disposition of patients with incidental PE, although the oncologists and other specialists are available for consultation. Essentially, all patients have scheduled follow-ups with oncologists or an easy referral path to other specialists.

#### Study sample

Eligible patients had cancer, were >18 years of age, and presented to the ED during the study period with a diagnosis of incidental PE found on routine contrast-enhanced chest CT performed for cancer staging. The CT scans were interpreted by board-certified radiologists. The CT scan, the interpretation of scan results, and the referral to the ED occurred on the same day. We defined incidental PE in accordance with the International Society on Thrombosis and Hemostasis definition: “PE identified in scans ordered primarily for staging or restaging of malignancy” [6], with the added requirement that the PE was not previously known to the patient and that the patient had not reported symptoms suggesting the possibility of a PE. We excluded patients who had a prior diagnosis of PE in the previous year or who were on anticoagulants at the time of presentation.

Patients were assessed at triage for chest pain, dyspnea, and unstable vital signs according to institutional best practices (heart rate >100 beats per minute, temperature >37.5 °C, oxygen saturation <93%, and systolic blood pressure <100 mmHg) and then evaluated by an ED physician. Routine practice for these patients included further evaluation with pulse oximetry during ambulation and blood testing (including platelets, coagulation studies, d-dimer, and renal function). The ED physician reviewed CT results, patient symptoms, vital signs, oxygen saturation, performance status, and comorbidities. Anticoagulation was initiated in the ED on the basis of the factors mentioned above and institutional best practices, which recommend low-molecular-weight heparin as the optimal treatment option for patients with cancer. Anticoagulation medication and dosage were determined on the basis of patient weight and clinician judgment.

Patients were admitted or discharged according to clinical assessment. Discharged patients and their caregivers were provided educational materials, including a PE information handout, and if applicable they were shown a video on injectable anticoagulants and were taught by ED nurses to administer the treatment. Discharged patients were given an outpatient appointment with internal medicine or their oncologist for follow-up, typically within 1 week. Email communication about the patient’s ED visit was sent to his or her oncologist.

### Study protocol

This research was conducted according to a clinical research protocol (DR08-0066) approved by the MD Anderson Institutional Review Board.

### Key outcome measures

The data abstractors (Srinivas Banala and Valda Page) were trained using a list of methodological evaluation criteria created by Gilbert et al. [28] and Worster et al. [29], to ensure that our methods remained consistent with these criteria. Data findings were independently reviewed by Terry Rice, and differences in opinion were refereed by Kumar Alagappan [28].

The primary outcome was 30-day survival from ED presentation for incidental PE. We used 30 days as a cutoff that is meaningful from the emergency physician perspective. The secondary outcome measures were 90-day survival and overall survival. These outcomes were analyzed for association with covariates such as PE location (described below), comorbidities, and functional status upon admission.

#### Demographic and clinical variables

Demographic variables, including age, sex, and race/ethnicity, along with certain clinical variables, such as cancer type and disease stage, were obtained from the MD Anderson Tumor Registry, which contains clinical and demographic data for every patient assigned a medical record number at MD Anderson along with follow-up information such as vital status, date of last contact/death, and method of follow-up—for example, phone calls, examination of other registries, and social security indices. Registry data are abstracted by trained and certified tumor registrars; quality control of abstracted information includes computerized edits for all applicable data items, and a second coder verifies neoplasm site, stage, and histology.

The location of the incidental PE within the pulmonary arteries was extracted from the CT report and was classified as saddle, main or lobar, segmental, or subsegmental, on the basis of the location of the clot most proximal to the heart.

Triage vital signs and other clinical data were documented in the ED and were extracted from the patient’s medical record for this study. Tachycardia was defined as a heart rate of >100 beats per minute. Hypotension was defined as a systolic blood pressure of <100 mmHg. Tachypnea was defined as a respiratory rate >20 breaths per minute. Hypoxemia was defined as oxygen saturation <93%. The disposition variable was based on whether or not the patient was admitted to the hospital from the ED.

Eastern Cooperative Oncology Group performance status (ECOG PS) [30] was part of our electronic health record history physical template and was recorded by ED physicians at the time of ED evaluation. ECOG PS is a standard, widely used clinician-rated criterion for measuring how disease affects a patient’s daily living and ability to function. A rating of 0 = patient is fully active, able to carry on all pre-disease performance without restriction; 1 = patient is restricted in physically strenuous activity but ambulatory, able to do light or sedentary work (e.g., light housework, office work); 2 = patient is ambulatory, capable of all self-care, up and about more than 50% of waking hours, but unable to carry out any work activities; 3 = patient is capable of only limited self-care, confined to bed or chair more than 50% of waking hours; 4 = patient is completely disabled, unable to carry on any self-care, totally confined to bed or chair.

Charlson comorbidity index (age unadjusted) scores were calculated from data collected in the chart review. The Charlson comorbidity index [31] predicts the risk of 1-year mortality in patients with a range of comorbid illnesses and has been validated in cancer patients. The index is based on the presence or absence of 17 comorbidities and assigns patients a score from 1 to 20, with 20 being the most complex cases having multiple comorbid conditions.

#### Survival data

Survival data was collected at 30 and 90 days from the date of ED presentation, as well as at the end of the study period. The cause of death was determined directly from the death summary if the patient had died at MD Anderson. For patients who were alive at each timepoint according to MD Anderson data, Tumor Registry representatives performed follow-ups to determine and record patient vital status (alive, or date, place, and cause of death).

### Data analysis

Descriptive statistics, including means, standard deviation (SD), and percentages, were used to summarize patient characteristics. Using the Kaplan–Meier method, we performed a univariate analysis of the association of patient characteristics with 30-day, 90-day, and overall survival. Multivariate Cox proportional hazard models were constructed on the basis of the univariate analysis and factors known to influence short-term survival (including PE location). Multivariate logistic regression analysis was used to assess the extent to which PE location influenced patient disposition (i.e., admission from the ED).

Statistical significance was set at *P* < 0.05. All statistical analyses were performed using SPSS software (SPSS Inc., Chicago, IL).

## Results

### Patient characteristics

Table 1 displays demographic and clinical characteristics of the patient sample, by disposition status. During the study period, 208 patients were sent to the ED after they were found to have incidental PE on a routine staging CT. Of these, 15 patients were excluded from analysis: 12 patients had recurrent PE, 1 had a benign tumor, and 2 were discharged against medical advice. Of the remaining 193 patients comprising the final sample, 111 (58%) were male and 82 (42%) were female; 139 (72%) were non-Hispanic white, 27 (14%) were African–American, 17 (9%) were Hispanic, and 10 (5%) were of another race/ethnicity. The mean age was 63 years (SD 12 years); 174 (90%) had solid tumors (exclusive of leukemia, lymphoma, myeloma, or stem cell transplant).

**Table 1:** Patient characteristics

Variable	Admitted (*n* = 58)	Discharged (*n* = 135)	*P*
Age, years, mean (SD)	65.23 (12.48)	61.51 (11.70)	0.756
Sex, no. (%)			0.641
Male	32 (55)	79 (59)	
Female	26 (45)	56 (41)	
Race/ethnicity, no. (%)			0.113
Non-Hispanic white	46 (79)	93 (69)	
Hispanic	2 (3)	15 (11)	
Black	9 (16)	18 (13)	
Other	1 (2)	9 (7)	
Cancer type, no. (%)			0.121
Liquid^a^	9 (16)	10 (7)	
Solid	49 (84)	125 (93)	
Cancer stage, no. (%)			0.086
IV	51 (88)	93 (69)	
III	3 (5)	20 (15)	
II	3 (5)	11 (8)	
I	1 (2)	11 (8)	
ECOG PS, no. (%)			0.009*
4	3 (5)	0 (0)	
3	6 (10)	7 (5)	
2	19 (33)	47 (35)	
1	28 (48)	69 (52)	
0	2 (3)	13 (10)	
Charlson comorbidity index (age unadjusted), mean (SD)	6.01 (1.63)	6.28 (1.33)	0.490
Tachycardia (>100 beats/min), no. (%)			0.008*
Yes	16 (28)	15 (11)	
No	42 (72)	120 (89)	
Hypotension (systolic <100 mmHg), no. (%)			0.610
Yes	1 (2)	2 (1)	
No	57 (98)	133 (99)	
Tachypnea (>20 breaths/min), no. (%)			0.739
Yes	2 (3)	5 (4)	
No	56 (97)	130 (96)	
Hypoxemia (oxygen saturation <93%), no. (%)			<0.001*
Yes	11 (19)	1 (1)	
No	46 (81)	134 (99)	
Incidental PE location, no. (%)			<0.001*
Saddle	7 (12)	0 (0)	
Main/lobar pulmonary artery	19 (33)	18 (13)	
Segmental	24 (41)	85 (63)	
Subsegmental	8 (14)	32 (24)	

*Abbreviations*: *ECOG PS* Eastern Cooperative Oncology Group performance status, *PE* pulmonary embolism, *SD* standard deviation

^a^Includes multiple myeloma, lymphoma, leukemia, stem cell transplant

*Significant at *P* < 0.05

### Outcomes

Overall, 16 of the 193 patients died within 30 days of ED presentation, representing an 8% mortality rate. Most (135 patients, 70%) were discharged, with 58 patients (30%) being admitted to the hospital. Almost all (189 patients, 98%) were started on anticoagulation therapy in the ED; 170 (90%) of these patients received low-molecular-weight heparin. The 4 patients who did not receive anticoagulation in the ED were either at high risk for bleeding or were actively bleeding and were admitted for inferior vena cava filter placement. Hospitalized patients were more likely than discharged patients to have tachycardia (16 patients [28%] versus 15 patients [11%]) and hypoxemia (11 patients [19%] versus 1 patient [1%]). Eight discharged patients experienced adverse events within 30 days of ED presentation: 3 had major bleeding, 3 had recurrent venous thromboembolism, and 2 died. Of the 2 discharged patients who died, 1 was recommended for hospice care, and the cause of death for the other is unknown. No treatment-related adverse events occurred in the admitted patients.

#### Survival outcomes

Of the 193 patients enrolled in the study, 96 patients had died and 97 patients had survived by the end of the study, for an overall survival rate of 51%. The 30-day survival rate was 92% overall, 76% for admitted patients and 99% for discharged patients. At 90 days, the survival rates were 84% overall, 69% for admitted patients and 90% for discharged patients.

We conducted multivariate analyses to assess the influences of various factors on survival 30 days from ED presentation, 90 days from ED presentation, and overall (Table 2). Charlson comorbidity index (age unadjusted), ECOG PS, hypoxemia, and incidental PE location were significantly associated with 30-day survival; Charlson comorbidity index (age unadjusted), cancer type, ECOG PS, and incidental PE location were significantly associated with 90-day survival. At the end of the study, cancer type, cancer stage, and ECOG PS were the only variables significantly associated with survival.

**Table 2:** Multivariate analysis of factors affecting survival outcomes

	30-day survival				90-day survival				Overall survival			
	*P*	RR	95% CL for RR		*P*	RR	95% CL for RR		*P*	RR	95% CL for RR	
			Lower	Upper			Lower	Upper			Lower	Upper
Age	0.272	0.975	0.932	1.020	0.768	0.995	0.962	1.029	0.388	1.009	0.989	1.029
Sex	0.248	0.455	0.120	1.731	0.262	0.623	0.272	1.425	0.431	0.837	0.537	1.304
Race (reference: Black)	0.724				0.858				0.949			
Hispanic	0.287	0.383	0.065	2.240	0.684	0.792	0.259	2.428	0.626	1.180	0.607	2.295
Other	0.697	1.532	0.178	13.152	0.485	0.484	0.063	3.704	0.809	0.911	0.430	1.933
Non-Hispanic white	0.972	0.000	0.000	undefined	0.654	0.696	0.143	3.385	0.808	1.129	0.422	3.021
Cancer type (liquid^a^ vs. solid)	0.955	0.000	0.000	undefined	0.018*	0.042	0.003	0.580	0.016*	0.218	0.063	0.752
Cancer stage (reference: 0)	0.941				0.938				0.004*			
I	>0.999	1.154	0.000		0.996	0.000	0.000		0.963	0.000	0.000	undefined
II	0.989	0.000	0.000		0.984	0.000	0.000		0.015	0.085	0.012	0.618
III	0.377	2.364	0.350	15.969	0.980	1.022	0.189	5.512	0.035	0.213	0.050	0.900
IV	0.961	0.000	0.000	undefined	0.375	0.501	0.109	2.306	0.013	0.363	0.163	0.809
ECOG PS (reference: 0)	0.136				0.084				0.020*			
1	0.972	0.000	0.000		0.979	0.000	0.000		0.010	0.084	0.013	0.553
2	0.013	0.028	0.002	0.473	0.093	0.145	0.015	1.384	0.190	0.416	0.112	1.543
3	0.079	0.099	0.008	1.308	0.311	0.328	0.038	2.836	0.192	0.427	0.119	1.536
4	0.135	0.106	0.006	2.004	0.824	0.773	0.081	7.419	0.910	1.085	0.263	4.475
Charlson comorbidity index (age unadjusted)	0.025*	0.518	0.291	0.922	0.017*	0.593	0.386	0.911	0.041*	0.766	0.592	0.990
Tachycardia (>100 beats/min)	0.714	0.718	0.122	4.235	0.635	0.761	0.246	2.356	0.804	1.081	0.582	2.008
Hypotension (systolic <100 mmHg)	0.995	103681.952	0.000		0.993	407878.592	0.000		0.710	1.465	0.195	10.983
Tachypnea (>20 breaths/min)	0.058	0.123	0.014	1.075	0.167	0.314	0.061	1.626	0.594	0.723	0.220	2.380
Hypoxemia (oxygen saturation <93%)	0.030*	0.096	0.011	0.800	0.207	0.385	0.088	1.694	0.978	0.987	0.388	2.510
Location of incidental PE (reference: subsegmental)	0.009*				0.083				0.170			
Saddle	0.004*	33.513	3.142	357.404	0.023	6.185	1.282	29.828	0.287	1.742	0.627	4.841
Main/lobar pulmonary artery	0.316	2.992	0.351	25.472	0.248	2.199	0.577	8.382	0.688	1.144	0.595	2.199
Segmental	0.713	1.492	0.177	12.549	0.570	1.425	0.420	4.826	0.269	0.736	0.428	1.267

*Abbreviations*: *CL* confidence limit, *ECOG PS* Eastern Cooperative Oncology Group performance status, *PE* pulmonary embolism, *RR* relative risk

^a^Includes multiple myeloma, lymphoma, leukemia, stem cell transplant

*Significant at *P* < 0.05

Risk for death within 30 days was higher if the incidental PE was located in the saddle pulmonary artery (43%). The multivariate analysis results reported in Table 2 indicate that the relative risk ratio for a subsegmental vs saddle incidental PE was 28.8 at 30 days, but that by 90 days it was only 7.0 (1.9 by the end of the study). Although statistically significant at 30 days, it was not statistically significant for overall survival; however, these groups may have been too small to achieve significance (only 7 patients with saddle and 40 with subsegmental).

#### Survival and disposition

Of the 16 patients who died within 30 days of ED presentation, 14 (88%) were admitted to the hospital, resulting in a 24% mortality rate for admitted patients. With regard to disposition, we found that age, Charlson comorbidity index (age unadjusted), race (non-Hispanic white or other), stage IV cancer, tachycardia, hypoxemia, and incidental PE location (main/lobar) were significantly associated (*P* < 0.05) with hospital admission (Table 3). Saddle and subsegmental PE location did not reach statistical significance in relation to hospital admission; however, as described above, these groups may have been too small to achieve significance.

**Table 3:** Multivariate logistic regression model of factors associated with hospital admission

	SE	Wald	*P*	OR	95% CL for OR	
					Lower	Upper
Age	0.023	4.556	0.033*	1.051	1.004	1.100
Sex	0.518	2.272	0.132	0.458	0.166	1.264
Race (reference: Black)		7.688	0.053			
Hispanic	0.710	0.194	0.659	0.731	0.182	2.943
Other	0.997	4.460	0.035	0.122	0.017	0.859
Non-Hispanic white	2.255	4.147	0.042	0.010	0.000	0.842
Cancer type (liquid^a^ vs. solid)	0.950	2.271	0.132	4.188	0.650	26.975
Cancer stage (reference: 0)		9.052	0.060			
I	40192.970	0.000	>0.999	0.000	0.000	
II	1.172	0.722	0.396	0.370	0.037	3.673
III	1.059	1.657	0.198	0.256	0.032	2.039
IV	1.319	7.336	0.007	0.028	0.002	0.373
ECOG PS (reference: 0)		5.537	0.237			
1	19503.505	0.000	0.999	0.000	0.000	
2	19503.505	0.000	0.999	0.000	0.000	
3	19503.505	0.000	0.999	0.000	0.000	
4	19503.505	0.000	0.999	0.000	0.000	
Charlson comorbidity index (age unadjusted)	0.209	0.308	0.579	0.891	0.592	1.341
Tachycardia (>100 beats/min)	0.643	5.249	0.022*	0.229	0.065	0.808
Hypotension (systolic <100 mmHg)	1.397	2.268	0.132	0.122	0.008	1.886
Tachypnea (>20 breaths/min)	1.402	1.126	0.289	4.427	0.284	69.090
Hypoxemia (oxygen saturation <93%)	1.116	11.110	0.001*	0.024	0.003	0.216
Location of incidental PE (reference: subsegmental)		18.526	<0.001*			
Segmental	14449.425	0.000	0.999	17913173674.808	0.000	
Main/lobar pulmonary artery	0.797	14.731	<0.001*	21.281	4.465	101.428
Saddle	0.628	1.120	0.290	1.943	0.568	6.645
Constant	19503.505	0.000	0.999	722285828219.644		

*Abbreviations*: *CL* confidence limit, *ECOG PS* Eastern Cooperative Oncology Group performance status, *OR* odds ratio, *PE* pulmonary embolism, *SE* standard error

^a^Includes multiple myeloma, lymphoma, leukemia, stem cell transplant

*Significant at *P* < 0.05

#### Effects of PE location and disposition

**Fig. 1:**
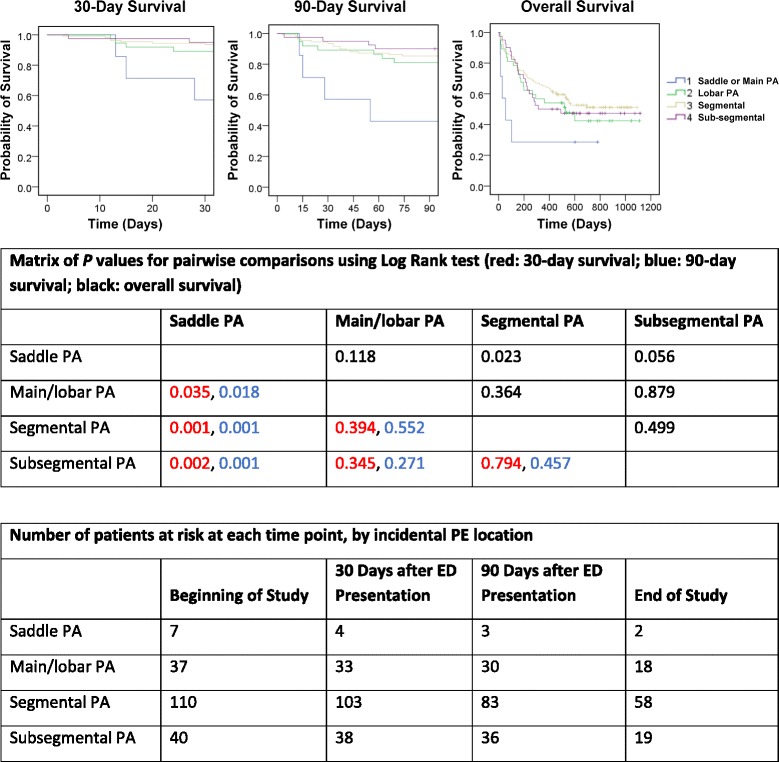
Kaplan–Meier curves showing the association between incidental PE location and survival. ED, emergency department PA, pulmonary artery

An incidental PE with saddle pulmonary artery location was found in 7 patients, all of whom were admitted; 3 died within 30 days of ED presentation (a 43% mortality rate). See Table 1. Main/lobar pulmonary artery PE was found for 37 patients; of these, 19 (51%) were admitted and 4 died within 30 days (an 11% mortality rate). Of the 109 patients with segmental PE, 24 (22%) were admitted and 7 of these died within 30 days (a 6% mortality rate). Of the 40 patients with subsegmental PE, 8 (20%) were admitted and 2 of these died within 30 days (a 5% mortality rate). See Fig. 1.

Of the 135 discharged patients, 2 (1%) died within 30 days of diagnosis. Of the 58 patients admitted to the hospital, 14 (24%) died within 30 days. Of the 16 patients who died within 30 days of diagnosis of incidental PE on CT, 19% had saddle PE, 25% main/lobar, 43% segmental, and 13% subsegmental.

## Discussion

Optimal management of an incidental PE is an important issue in the emergent care of the patient with cancer, yet to our knowledge, no standard guidelines exist to direct ED treatment teams in the management and disposition of patients with incidental PE who also have cancer. Management decisions should be framed within an understanding of the short-term and long-term prognoses of these patients: considering the intensive treatment regimens, diminished quality of life, and possible mortality faced by patients with cancer, along with an oft-expressed desire to be home in their last days, respectful management of their time by avoiding unnecessary hospitalization could not be more important. These decisions are made more difficult by the lack of research that includes cancer patients and by persistent descriptions of them as “high risk.” The results of this study suggest that selected patients treated with anticoagulation in an outpatient setting may be at low risk for adverse events, given that only 2 of 135 outpatients in our sample (one of whom had been recommended to hospice) died within 30 days of ED presentation.

We selected 30-day, 90-day, and overall (end of study) timepoints to mimic the natural history of PE. Our expectation was that mortality and other adverse events closer to ED presentation (30 days) would more likely indicate that the PE might be relevant to those outcomes, whereas events more distal to the ED presentation (90 days and beyond) would be less likely to be related to the PE. In the multivariate analysis of factors affecting survival outcomes, the subsegmental versus saddle location of the PE was no longer statistically significant by end of study, possibly indicating that in the long term, underlying disease and comorbidities were the main factors affecting survival in treated patients.

In a recent study by Singer et al. [32], it was noted that from 2006 to 2010, the admission rate for patients in US EDs with PE was 90%, without any significant change over that time period. It is likely that physicians are reluctant to treat cancer patients with PE as outpatients, owing to uncertainty on how to safely identify those individuals who are at low risk for short-term adverse events, irrespective of whether the adverse events could be averted by hospitalization. Existing point-based risk-stratification systems that assign a point for having cancer would automatically place cancer populations into the high-risk group and therefore would not be applicable here. Nonetheless, recent studies have suggested that outpatient management in a selected group of patients with acute PE and stable hemodynamic status is safe [10, 33, 34].

In our study, patients who were admitted to the hospital (*n* = 58; 30%) were more likely to die than were discharged patients (*n* = 135; 70%), as would be expected. These results were similar to those from a cohort study by Erkens et al. [33], who used a like process to determine whether patients with suspected (nonincidental) PE could be managed as outpatients instead of being hospitalized (i.e., patient is hemodynamically stable, does not require supplemental oxygenation, has no contraindications to low-molecular-weight heparin, and no significant comorbidities; ultimately decided by the ED doctor based on his or her clinical judgment). The rate of serious adverse events in that study was higher in the admitted patients, which, taken together with our results, suggests that simple criteria can be used to discriminate between low-risk and high-risk patients.

Further work that standardizes the evaluation and management of these patients and a prospective application of such guidelines would provide improved guidance. In the current study, 170 patients (88%) were started on low-molecular-weight heparin; this practice is consistent with several trials that reported low-molecular-weight heparin monotherapy to be more effective than conventional treatment with vitamin K antagonists for the long-term management of cancer-associated venous thromboembolism. Nonetheless, determination of the optimal pharmaceutical management of this patient group is limited by the small number of patients available at the institutional level, highlighting the need for more research and collaboration among those who provide emergency care to cancer patients. In addition, further work needs to be done to determine if additional lives could be saved with more aggressive intervention, such as systemic or intravascular thrombolysis, or if quality of life or cost effectiveness could be improved through the use of oral agents.

Limitations include the fact that this study was conducted in a single center with a limited number of patients, and it was retrospective in nature, with no randomization of treatment. Further, the health outcomes related to PE are not well distinguished from those related to advanced cancer, further limiting the generalizability of the results. Our findings should be corroborated in a larger randomized, controlled clinical trial. The retrospective nature of the study and single center make it difficult to translate our results to other settings, especially those with less cancer expertise. However, our purpose in conducting this study was to disseminate knowledge gained at a comprehensive cancer center in a useful way, so that treatment teams at EDs with smaller cancer populations might be able to quickly assimilate these considerations.

## Conclusions

Our results suggest that selected cancer patients presenting to the ED with incidental PE who resemble the discharged patients in our cohort (without hypoxemia, significant comorbidities, or saddle PE) can be given anticoagulation therapy with low-molecular-weight heparin and discharged safely for outpatient management. Prospective research to verify these results in an array of ED practice settings (e.g., non-cancer centers, community EDs) is needed, however, to solidify this conclusion. The implications of this line of research are wide ranging, as avoidance of unnecessary hospitalization may decrease in-hospital infections and death, reduce healthcare costs, and improve patient quality of life [35–37].

## Abbreviations

CL: Confidence limit
CT: Computed tomography
ECOG PS: Eastern Cooperative Oncology Group performance status
ED: Emergency department
PA: Pulmonary artery
NIH: National Institutes of Health
OR: Odds ratio
PE: Pulmonary embolism
RR: Relative risk
SD: Standard deviation
SE: Standard error
